# The Dissemination of Intra-Abdominal Malignant Disease by Means of the Lymphatics and Theoracic Duct

**Published:** 1907-05

**Authors:** 


### THE DISSEMINATION OF INTRA-ABDOMINAL MALIGNANT DISEASE BY MEANS OF THE LYMPHATICS AND THORACIC DUCT.

---

The author, W. M. Stevens, has made a very careful antomical study of the lymphatic supply and drainage of the upper abdomen, and reports 7 clinical observations that bear out his conclusions. Ther are:

The thoracic duct undoubtedly plays an important role in the dissemination of intra-abdominal malignant disease and of tuberculosis. This duct may act as a "simple carrier" of infective material or it may be directly involved, and in some cases it may be actually obstructed. In connection with blocking of this duct it is very remarkable that chylous ascites is so rare and also that dilatation of other lymphatic channels can so seldo mbe demonstrated.

The supraclavicular glands on the left side are more frequently involved in intramalignant disease than is generally supposed, and in many cases careful percussion will show the presence of glandular enlargement in the clavicular and infraclavicular regions, and may even thus give a clue to the nature of the abdominal affection. It is possible that these glands become infected through "regurgitation," but a more likely method of infection, and one which can be demonstrated in some cases, is by direct communication of the disease along the walls of the thoracic duct, extending to the lymphatic vessel coming from these glands. The right clavicular glands are very seldom involved, and the reason for this is fairly obvious on studying the lymphatic anatomy.

The possible mode of infection of other parts—chest, liver, inguinal glands, abdominal wall, etc.—have been discussed.

It is probable that many cases of so-called primary mediastinal growths may really be of secondary origin, since it is well known that cancerous disease, especially of the stomach, may be very "latent," and, moreover, a small growth of the stomach, though it has been the cause of marked secondary growth, may even be overlooked at the autopsy unless especial care be taken.—British Med. Jour., Feb. 9th, '07.

